# Leveraging protein language models for cross-variant CRISPR/Cas9 sgRNA activity prediction

**DOI:** 10.1093/bioinformatics/btaf385

**Published:** 2025-07-02

**Authors:** Yalin Hou, Yiming Li, Ruiqing Zheng, Fuhao Zhang, Fei Guo, Min Li, Min Zeng

**Affiliations:** School of Computer Science and Engineering, Central South University, Changsha 410083, China; School of Computer Science and Engineering, Central South University, Changsha 410083, China; School of Computer Science and Engineering, Central South University, Changsha 410083, China; College of Information Engineering, Northwest A&F University, Yangling, Shaanxi 712100, China; School of Computer Science and Engineering, Central South University, Changsha 410083, China; School of Computer Science and Engineering, Central South University, Changsha 410083, China; School of Computer Science and Engineering, Central South University, Changsha 410083, China

## Abstract

**Motivation:**

Accurate prediction of single-guide RNA (sgRNA) activity is crucial for optimizing the CRISPR/Cas9 gene-editing system, as it directly influences the efficiency and accuracy of genome modifications. However, existing prediction methods mainly rely on large-scale experimental data of a single Cas9 variant to construct Cas9 protein (variants)-specific sgRNA activity prediction models, which limits their generalization ability and prediction performance across different Cas9 protein (variants), as well as their scalability to the continuously discovered new variants.

**Results:**

In this study, we proposed PLM-CRISPR, a novel deep learning-based model that leverages protein language models to capture Cas9 protein (variants) representations for cross-variant sgRNA activity prediction. PLM-CRISPR uses tailored feature extraction modules for both sgRNA and protein sequences, incorporating a cross-variant training strategy and a dynamic feature fusion mechanism to effectively model their interactions. Extensive experiments demonstrate that PLM-CRISPR outperforms existing methods across datasets spanning seven Cas9 protein (variants) in three real-world scenarios, demonstrating its superior performance in handling data-scarce situations, including cases with few or no samples for novel variants. Comparative analyses with traditional machine learning and deep learning models further confirm the effectiveness of PLM-CRISPR. Additionally, motif analysis reveals that PLM-CRISPR accurately identifies high-activity sgRNA sequence patterns across diverse Cas9 protein (variants). Overall, PLM-CRISPR provides a robust, scalable, and generalizable solution for sgRNA activity prediction across diverse Cas9 protein (variants).

**Availability and implementation:**

The source code can be obtained from https://github.com/CSUBioGroup/PLM-CRISPR.

## 1 Introduction

The CRISPR–Cas9 system has revolutionized the life sciences (Allemailem *et al.* 2022, Wang and Doudna 2023), providing a powerful tool for genome editing (Zhu *et al.* 2020). Originating from bacterial immune systems, CRISPR–Cas9 uses the Cas9 nuclease guided by a single-guide RNA (sgRNA) to target specific DNA sequences (Li *et al.* 2023). The sgRNA guides Cas9 to the target site, where it recognizes a protospacer adjacent motif (PAM) and introduces precise double-strand breaks (Farboud *et al.* 2018). This targeting mechanism relies on the complementarity between the sgRNA's 20 nucleotide sequence and the target DNA (Komor *et al.* 2016). Due to its remarkable efficiency and adaptability, CRISPR–Cas9 has significantly advanced gene-editing technologies (Gaudelli *et al.* 2017), highlighting the critical importance of accurately predicting sgRNA activity to optimize its application.

Traditional approaches for designing and selecting highly efficient sgRNAs typically rely heavily on experimental screening, which is both time-consuming and labor-intensive (Xiang *et al.* 2021). To address these limitations, computational models for predicting sgRNA activity have gained significant attention, aiming to offer a more efficient and cost-effective strategy for optimizing sgRNA selection (Anzalone *et al.* 2019). Existing sgRNA activity prediction methods can be broadly classified into three categories: alignment-based methods (Bae *et al.* 2014), rule-based methods (Heigwer *et al.* 2014, Montague *et al.* 2014), and learning-based methods (machine learning/deep learning) (Wong *et al.* 2015, Doench *et al.* 2016). Alignment-based methods analyse the similarity between sgRNA and target DNA by sequence alignment tools (e.g. BLAST), but they are prone to rely too much on sequence similarity, leading to the neglect of other influencing factors, and they have high computational resource requirements. Rule-based methods rely on rules summarized by expert knowledge to extract sgRNA sequence features (e.g. PAM sequences, avoidance of specific structures), and use traditional machine learning models (e.g. SVM, logistic regression) to construct models. However, there are limitations such as relying on expert knowledge, incomplete feature extraction, and generalization ability. Alignment-based and rule-based methods use predefined sequence features or rules to guide the design, thus their prediction performance is usually limited (Zhu *et al.* 2014, Liu *et al.* 2015). In recent years, learning-based methods have emerged as the dominant methods, leveraging advanced sequence representations and feature extraction techniques to learn significant patterns and improve prediction performance. For instance, sgRNACNN uses convolutional neural networks (CNNs) to capture local sequence features, demonstrating strong performance in large-scale genome editing (Niu *et al.* 2021). CRISPRON integrates the gRNA-target-DNA binding energy features to enhance CRISPR–Cas9 gRNA efficiency prediction performance (Xiang *et al.* 2021). DeepCRISPR employs deep convolutional denoising autoencoders to learn sgRNA sequence representations (Chuai *et al.* 2018). DeepHF uses RNN to capture the order and contextual relationships between individual bases in sgRNA sequences to model sequence long-term dependencies (Wang *et al.* 2019). C-RNNCrispr combines CNNs with RNNs to capture both local and long-range dependencies (Zhang *et al.* 2020). CRISPRONT integrates attention mechanisms with CNNs to capture the intrinsic characteristics of Cas9–sgRNA binding and cleavage (Zhang *et al.* 2021).

Despite significant advancements in sgRNA activity prediction, several critical limitations remain unresolved. First, most existing models are trained on large-scale datasets specific to a single Cas9 variant (e.g. SpCas9 (Kim and Kim 2014)), which limits the generalizability and applicability to other Cas9 variants. Second, although some methods employ transfer learning or joint training strategies to address new variant with limited datasets, they remain confined to specific variants and fail to adapt effectively to novel ones. Third, existing models typically focus on sgRNA sequence features while ignore the critical role of Cas proteins variants in the editing system. As a result, these models struggle to achieve accurate predictions across multiple variants and lack the flexibility and scalability required for novel variant applications.

With the continuous emergence of new CRISPR–Cas9 variants, the development of computational models capable of generalizing across these variants has become increasingly urgent (Li *et al.* 2024). A major challenge in developing a generalizable cross-variant framework is that the mutation sites among different variants are very few and sparsely distributed, making it difficult to effectively characterize and capture the differences between different variants. However, recent breakthroughs of pre-trained large language models (LLMs) on various bioinformatics tasks (Lin *et al.* 2023, Liu *et al.* 2024, Xiao *et al.* 2025, Zeng *et al.* 2025) have demonstrated their powerful contextual representation abilities, offering a promising opportunity for solving the problem of characterizing Cas9 protein (variants).

Inspired by the success of LLMs, we propose PLM-CRISPR, a novel dual-input deep learning framework designed to predict sgRNA activity across both established and novel variants by integrating sgRNA and Cas9 protein (variants) characteristics. PLM-CRISPR leverages a pre-trained protein language model (PLM), ESM2, to generate rich, contextual representations of Cas9 protein (variants), and uses a dynamic weight fusion module to combine the refined representations of protein and sgRNA sequences, thereby effectively capturing the interaction patterns between them.

Experimental results demonstrate that the cross-variant training strategy employed by PLM-CRISPR significantly improves sgRNA activity prediction performance compared to traditional variant-specific methods. Extensive benchmarking demonstrates that PLM-CRISPR consistently outperforms existing sgRNA activity prediction methods, traditional machine learning methods, and deep learning methods across datasets spanning seven Cas9 protein (variants). Furthermore, we demonstrate PLM-CRISPR’s superior performance in three real-world scenarios, exhibiting its remarkable robustness in small-sample learning and its superior generalization capabilities across diverse CRISPR/Cas variants.

## 2 Materials and methods

### 2.1 Data collection and processing

#### 2.1.1 Multi-variant sgRNA activity data collection

**Figure 1.**
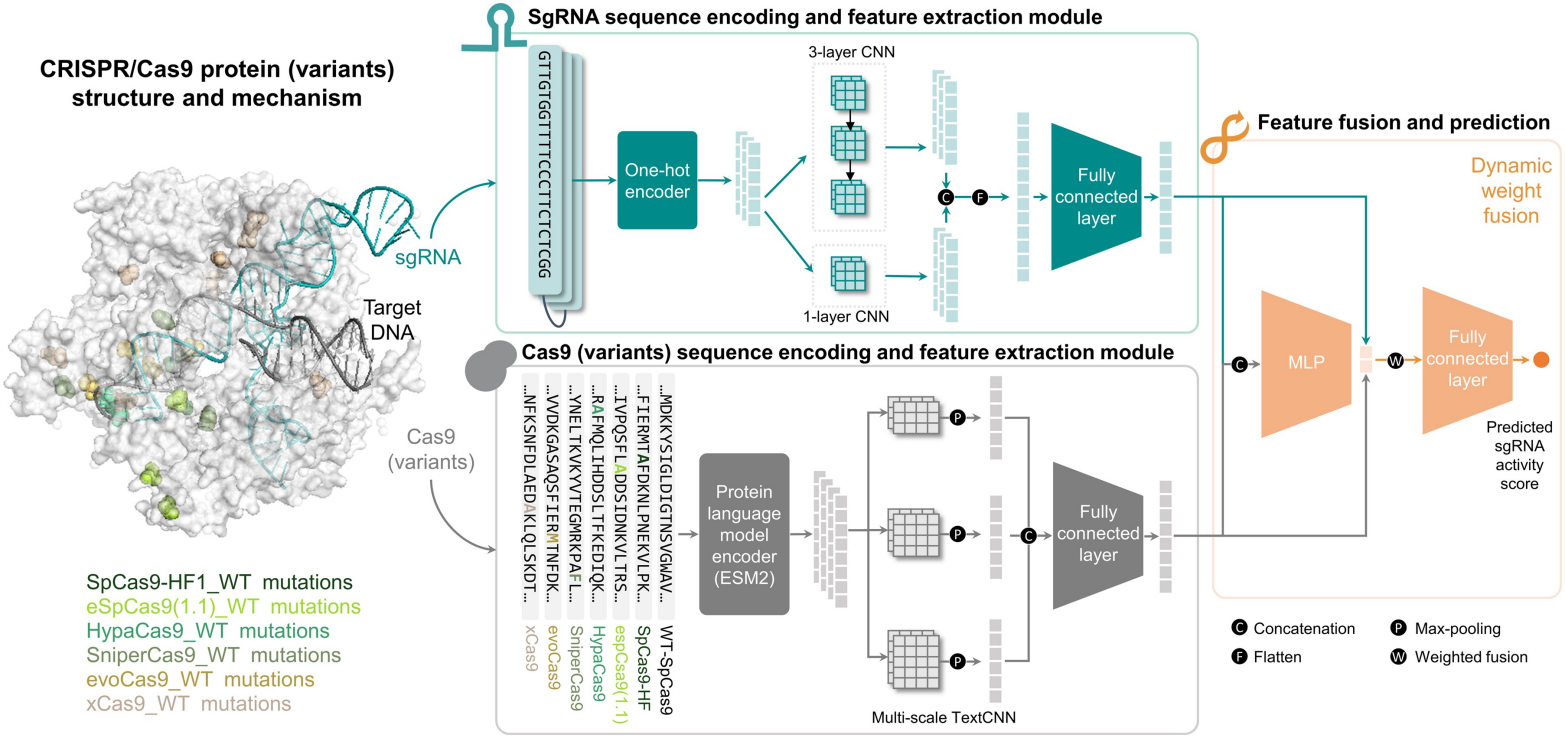
Overview of the PLM-CRISPR framework for predicting sgRNA activity across Cas9 protein (variants). The framework takes two types of input: the sgRNA sequence and the protein variant sequence. Each type of biological data undergoes specialized preprocessing modules. For sgRNA sequences, one-hot encoding is used to generate initial representations, which are then processed by multi-layer CNNs for feature extraction. Cas9 protein (variants) sequences are encoded using PLM ESM2 and further processed through a TextCNN. The features from both paths are dynamically weighted and integrated for the final classification. The “SpCas9-HF1_WT mutations” in the left panel refers to the mutation sites of the Cas9 variant SpCas9-HF1 relative to the wild-type *Streptococcus pyogenes* Cas9 (WT-SpCas9), as is the case for the other variants.

To construct sgRNA activity datasets across different Cas9 protein (variants) for model training and evaluation, we collected and processed data from multiple sources. Specifically, we collected datasets containing only the NGG PAM sequence from Xiang *et al.* (2021), Kim *et al.* (2020), and Wang *et al.* (2019). We removed missing values and eliminated duplicates within each dataset, resulting in approximately 180 000 samples spanning seven Cas9 protein (variants). Each sample consists of a 59-nt RNA sequence, including a 20-nt protospacer, a 3-nt PAM, and a 36-nt scaffold sequence, along with its corresponding activity value. Notably, the former 23-nt region of the sgRNA sequence plays a primary role in determining the efficiency and specificity of sgRNA activity. The processed datasets were partitioned into training, validation, and test sets in a 7:1:2 ratio, as detailed in Table 1. The histograms of sgRNA activity scores for the training, validation, and test sets across all datasets are shown in Fig. 1, available as supplementary data at *Bioinformatics* online.

**Table 1.** Details of sgRNA activity datasets across different Cas9 protein (variants).

Datasets	Cell lines	Cas9 protein (variants)	Training samples	Validation samples	Testing samples	Total	Dataset source
WT_kim	HEK293T	WT-SpCas9	3884	553	1109	5546	Kim *et al.* (2020)
HF_kim	HEK293T	SpCas9-HF1	3935	564	1132	5631	Kim *et al.* (2020)
esp_kim	HEK293T	eSpCas9 (1.1)	3952	567	1129	5648	Kim *et al.* (2020)
WT_wang	HEK293T	WT-SpCas9	32246	4606	9246	46098	Wang *et al.* (2019)
HF_wang	HEK293T	SpCas9-HF1	32975	4711	9473	47159	Wang *et al.* (2019)
esp_wang	HEK293T	eSpCas9 (1.1)	34030	4844	9723	48597	Wang, *et al.* (2019)
WT_xiang	HEK293T	WT-SpCas9	6188	884	1768	8840	Xiang *et al.* (2021)
Hypa	HEK293T	HypaCas9	823	117	235	1175	Kim *et al.* (2020)
sniper	HEK293T	SniperCas9	963	137	275	1375	Kim *et al.* (2020)
evo	HEK293T	evoCas9	949	135	271	1355	Kim *et al.* (2020)
xcas9	HEK293T	xCas9	955	137	273	1365	Kim *et al.* (2020)

#### 2.1.2 Variant protein sequence generation

We generated the amino acid sequences of the seven Cas9 variants involved in this study by mutating the parental sequence. All Cas9 protein (variants) possess an amino acid sequence length of 1368. The mutation sites for each Cas9 variant were obtained from Addgene’s plasmid records (Kamens 2015) and further validated through relevant literature, as shown in Table 2. The detailed information and functions of the key domains involved at the mutation sites of the Cas variants are shown in Table 1, available as supplementary data at *Bioinformatics* online. The parental sequence is the wild-type Cas9 (WT-SpCas9) from *Streptococcus pyogenes*, sourced from the UniProt database (Consortium 2007). Using the parental sequence as a template, mutations were applied sequentially (e.g. R661A indicates the substitution of arginine at position 661 with alanine). To ensure consistency with published data, we verified the variant sequences by aligning them with Addgene’s reference sequences using the CLUSTALW tool (Thompson *et al.* 1994).

**Table 2.** Mutation site details of Cas9 protein (variants).

Cas9 protein (variants)	Mutation sites	Source
WT-SpCas9	————	Jinek *et al.* (2012)
SpCas9-HF1	N497A, R661A, Q695A, Q926A	Kleinstiver *et al.* (2016)
eSpCas9(1.1)	K848A, K1003A, R1060A	Slaymaker *et al.* (2016)
HypaCas9	N692A, M694A, Q695A, H698A	Chen *et al.* (2017)
SniperCas9	F539S, M763I, K890N	Lee *et al.* (2018)
evoCas9	M495V, Y515N, K526E, R661Q	Casini *et al.* (2018)
xCas9	A262T, R324L, S409I, E480K, E543D, M694I, E1219V	Hu *et al.* (2018)

### 2.2 PLM-CRISPR architecture

In CRISPR systems, the efficacy of sgRNA directly impacts the precision and efficiency of gene editing, while variations in Cas9 protein (variants) can alter the binding efficiency between sgRNA and the target DNA sequence. Accordingly, our model design extends beyond analysing sgRNA sequences alone by incorporating protein variant sequences as essential contextual information. This dual-input approach aims to comprehensively leverage and integrate features from both sgRNA and protein variant sequences to improve the prediction performance of sgRNA activity. As illustrated in Fig. 1, PLM-CRISPR is a deep learning model comprising three modules: (i) sgRNA sequence encoding and feature extraction module, (ii) Cas9 protein (variants) sequence encoding and feature extraction module, and (iii) feature fusion and prediction module. All the symbols involved in the formulas of each module, along with their definitions, are presented in Table 2, available as supplementary data at *Bioinformatics* online.

#### 2.2.1 sgRNA sequence encoding and feature extraction

For sgRNA sequences, we employed one-hot encoding for the four nucleotides (A, C, G, T) and the special nucleotide N. This widely used encoding method converts RNA sequences into numerical matrices in the sgRNA activity prediction task. Specifically, it represents the input 59-nt-long sequence as a 59 × 5 one-hot embedding matrix.

CNN models are one of the most powerful deep learning frameworks for biological sequences. They have been successfully applied to RNA-related research, demonstrating exceptional capability in extracting local and deep feature information from RNA sequences. In order to capture richer multi-scale features from sgRNA sequence vectors, we used a dual-path CNN module. The dual-path CNN module has two branches that separately extract high-dimensional features from sgRNA sequences. Specifically, the dual-path CNN module consists of a single-layer convolutional layer, and a three-layer convolutional layer. Each convolutional layer applies a kernel size of 5 and a padding size of 2. The single convolutional layer in the first path is formulated as follows:


(1) $$\mathrm{Con}v_{\mathrm{single}}{\left(X_{\mathrm{RNA}}\right)}_{i,k}=\sigma (\sum_{m=0}^{M-1}\;\sum_{n=0}^{N-1}\;W_{k}X_{i+m,n}+b_{k})$$

where $X_{\mathrm{RNA}}\in R^{L\times D}$ denotes the input matrix of the sgRNA sequence, L denotes the length of the input sequence, D denotes the embedding dimension of the input, i denotes the output position index, k denotes the index of the filter, M denotes the window size, N denotes the number of input channels, $W_{k}$ denotes the convolutional filter, $b_{k}$ denotes the bias of the kth convolution kernel, and σ denotes the Sigmoid activation function. The three-layer convolution in the second path is defined as follows:


(2) $$\mathrm{Con}v_{\mathrm{three}}{\left(X_{\mathrm{RNA}}\right)}_{i,k}=\sigma (\sum_{m=0}^{M-1}\;\sum_{n=0}^{N^{\left(2\right)}-1}\;W_{k}^{\left(l\right)}Z_{i+m,n}^{\left(2\right)}+b_{k}^{\left(3\right)})$$

(3) $$Z_{i,k}^{\left(l\right)}=\sigma (\sum_{m=0}^{M-1}\;\sum_{n=0}^{N^{\left(l\right)}-1}\;W_{k}^{\left(l\right)}Z_{i+m,n}^{\left(l-1\right)}+b_{k}^{\left(l\right)}),l=1,2$$

(4) $$Z_{i,k}^{\left(0\right)}={X_{\mathrm{RNA}}}_{i,k}$$

where l denotes the lth convolutional layer, $Z^{\left(l\right)}$ denotes the output features of the lth layer. Following the convolutional layers, the extracted features from both paths adopt a shape of 59 × 64. Then, they are concatenated, flattened, and fed into a fully connected layer with 2048 neurons. The output is subsequently projected into a 256-dimensional vector via a linear layer for final feature fusion and prediction.

#### 2.2.2 Cas9 protein (variants) sequence encoding and feature extraction

For protein variant sequences, we used ESM2 (Verkuil *et al.* 2022), a large PLM that incorporates evolutionary information through multiple sequence alignments, to generates amino acid sequence embedding. ESM2 represents each amino acid in a protein sequence as a 1280-dimensional feature vector, capturing rich contextual and evolutionary information essential for downstream tasks.

Although Cas9 protein (variants) share high sequence similarity, the differences at key mutation sites can significantly influence sgRNA activity. These mutations often located in critical regions of the protein, such as the conserved RuvC and HNH domains, which can alter the interaction between sgRNA and the target DNA, thus affecting the efficiency and specificity of gene editing. To capture these crucial region features, we used a multi-scale TextCNN architecture to efficiently extract these conserved local patterns, which has demonstrated effectiveness in recent Cas protein identification studies (Yan *et al.* 2025). The multi-scale TextCNN employs three distinct kernel scales to extract potential useful local features from protein sequences. Smaller kernel scales focus on fine-grained details, while larger kernel scales detect broader patterns. Each scale contains 128 kernels to generate rich feature maps from the ESM2 encoded protein sequence feature matrix. This operation can be represented as follows:


(5) $$\mathrm{Con}v_{pt}^{\left(j\right)}{\left(X_{pt}\right)}_{i,k}=\mathrm{ReLU}(\sum_{m=0}^{M_{j}-1}\;\sum_{n=0}^{N-1}\;W_{k}^{\left(j\right)}\cdot {X_{pt}}_{i+m,n}+b_{k}^{\left(j\right)})$$

where $X_{pt}\in R^{L\times D}$ denotes the input matrix of protein sequences, j denotes the scale index, $M_{j}\in \{5,\;9,\;13\}$ denotes the kernel size of the jth scale, and ReLU denotes the rectified linear activation function.

After the convolution operation, we applied GlobalMaxPool1D to further extract the most prominent local features from each feature map


(6) $$Z_{pt}^{\left(j\right)}(X_{pt})=\mathrm{maxCon}v_{pt}^{\left(j\right)}{\left(X_{pt}\right)}_{i,k}$$

Finally, the obtained multi-dimensional features of different scales are flattened and then fed into a fully connected layer to obtain the output. The computation formulas can be represented as follows:


(7) $$H_{pt}=\sigma (W\cdot \mathrm{Concat}[Z_{pt}^{\left(j\right)}(X_{pt})]+b),j=1,2,3$$

This approach effectively extracts key local patterns while mitigating the impact of irrelevant noise, thereby effectively alleviating overfitting issues. To integrate global features, the multi-dimensional features obtained through convolutional layers and max pooling layers are flattened and fed into a fully connected layer, providing a comprehensive representation of the protein sequence for subsequent processing steps.

#### 2.2.3 Feature fusion and prediction

To integrate the processed sgRNA and protein features, we employed a dynamic weight fusion module. This module employs a weight network to dynamically adjust the contribution ratios of the sgRNA and protein features, generating a final comprehensive feature representation. This adaptive mechanism allows the model to flexibly balance the importance of each feature across different samples, enabling it to better accommodate the diversity and complexity of the data. The implementation of dynamic weight fusion consists of three main steps: feature concatenation, weight network computation, and feature weighted fusion.

- Feature concatenation: The sgRNA features and protein features are concatenated along the feature dimension to generate a unified feature vector:
  (8) $$Z_{\mathrm{combined}}=\mathrm{Concat}[H_{\mathrm{RNA}},H_{pt}]$$
  where $H_{\mathrm{RNA}}$ denotes the output of the sgRNA sequence feature extraction module, $H_{pt}$ denotes the output of the protein variant sequence feature extraction module.
- Weight network computation: a multi-layer perceptron (MLP) is used to compute the dynamic weights for each sample. This network consists of two linear layers and a sigmoid activation function, ensuring that the generated weights sum to 1. The weight computation is defined as:
  (9) w=σ(W(2)⋅ReLU(W(1)⋅Zcombined+b(1))+b(2))
  where w denotes the weight of the sgRNA feature while 1 − w denotes as the weight for the protein feature.
- Feature weighted fusion: the weight w is used to perform weighted fusion of the feature vectors, yielding the final comprehensive feature representation:
  (10) $$H_{\mathrm{fusion}}=w\cdot H_{\mathrm{RNA}}+(1-w)\cdot H_{pt}$$

Finally, the fused features are passed through two fully connected layers followed by a sigmoid activation function to predict sgRNA activity.

### 2.3 Implementation details

PLM-CRISPR was implemented using Python 3.8 and PyTorch 2.0.1. The experiments were conducted on two NVIDIA GeForce RTX 3090 GPUs, each equipped with 24 GB of memory, for both training and testing. PLM-CRISPR uses a grid search strategy to find the best combination of hyperparameters. In this study, the value of learning rate is chosen from {0.0001, 0.0005, 0.001, 0.005}, batch size is chosen from {64, 128, 256, 512}, and the epoch number is chosen from {50, 100, 200}. Finally, the optimal values for each hyperparameter combination setting are in Table 3. Mean squared error was employed as the loss function for the regression task, while the Adam optimizer was used as the optimizer to minimize the loss function and optimize the model parameters.

**Table 3.** The optimal hyperparameter of PLM-CRISPR.

Hyperparameter	Value
Learning rate	0.0001
Batch size	256
Number of epochs	100
Optimizer	Adam
Loss function	MSE
SgRNA feature extraction module kernel scales	64
Cas9 feature extraction module kernel scales	[5, 9, 13]
Learning rate scheduler	Cosine annealing
Learning rate scheduling cycle	5

To quantitatively evaluate the performance of PLM-CRISPR, we utilized the Spearman correlation coefficient (ρ) as the primary metric. This metric measures the alignment between the rank-order relationships of predicted sgRNA activity scores and the ground truth activity order. The Spearman correlation coefficient is calculated using the following formula:

(11) $$\rho =1-\frac{6\sum_{i=1}^{I}\;{d_{i}}^{2}}{n(n^{2}-1)}$$

where I denotes the total number of observations and $d_{i}$ denotes the rank difference of the ith data point between the predicted and true values.

## 3 Results

### 3.1 Comparison between variant-specific and cross-variant training strategy

**Figure 2.**
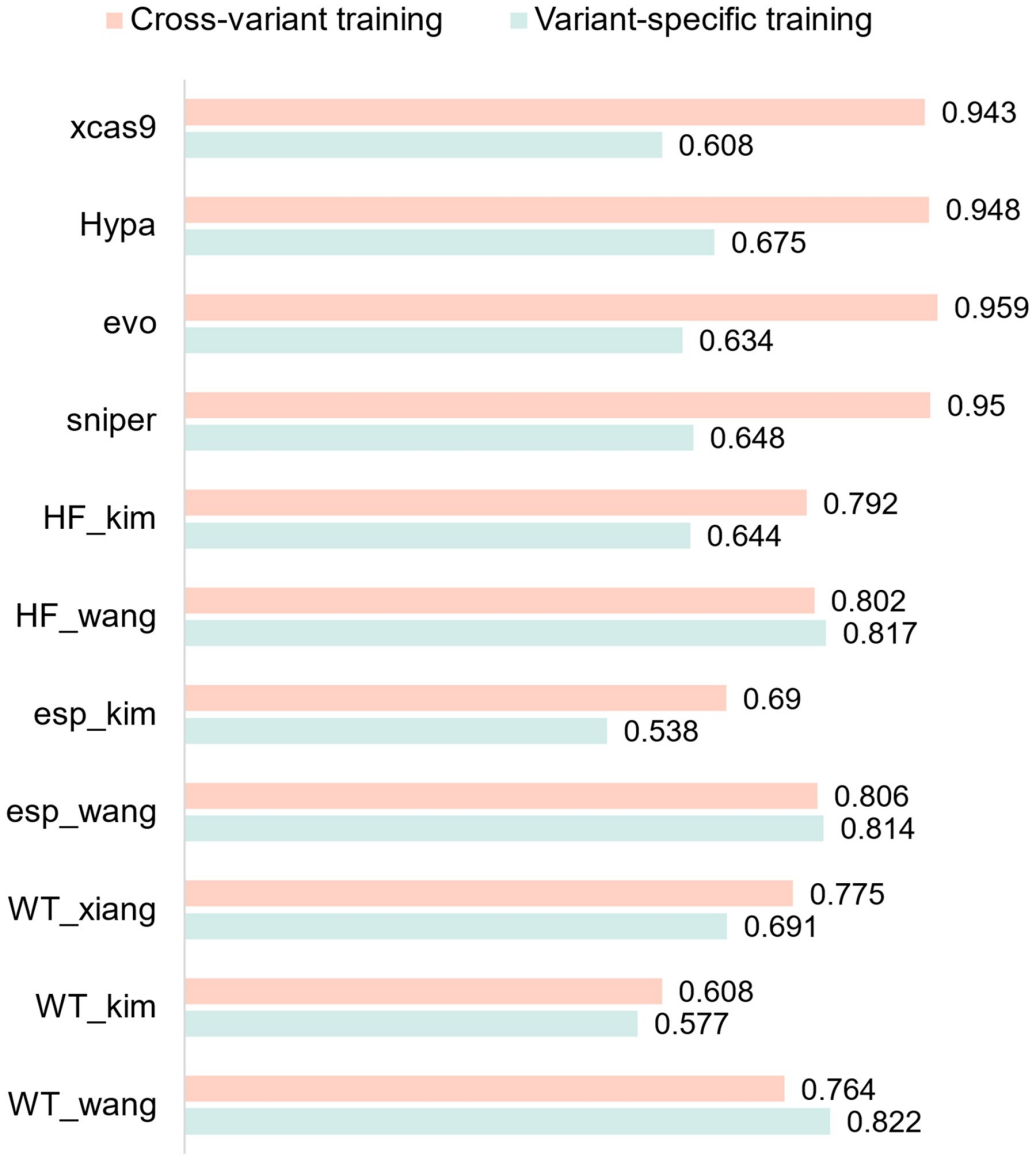
Comparison of Spearman correlation coefficients between variant-specific training and cross-variant training strategy for each dataset.

To demonstrate the superiority of the cross-variant training strategy over the traditional variant-specific model training settings, we conducted a comprehensive comparison between the two strategies. Variant-specific training strategy focuses on training a dedicated model for each individual Cas9 protein (variants) dataset. Accordingly, 11 separate models were trained on the 11 collected datasets. In contrast, the cross-variant training strategy integrates data of multiple protein variants to train a single universal model. As shown in Fig. 2, the cross-variant training strategy outperformed the variant-specific approach in eight out of 11 datasets. Notably, for the Sniper dataset, the Spearman correlation coefficient increased from 0.648 to 0.950, exhibiting a significant improvement of 46.6%. The variant-specific models rely solely on sgRNA features, which increases the risk of overfitting and limits their ability to generalize across variants. This approach often performs poorly on small or specific datasets. Figure 2 shows that the variant-specific models perform slightly better than the cross-variant models in the three datasets (esp_wang, HF_wang, and WT_wang). This is likely due to their larger sample sizes (over 40 000 sgRNA activity records), which provided sufficient training data for these models to learn variant-specific features thoroughly. Conversely, for smaller datasets, the cross-variant model performed better as it could leverage data from other large-scale datasets to enhance its generalization ability on small-scale datasets.

In contrast, the cross-variant training strategy aggregates data from multiple variants, building a more comprehensive feature space. This not only enhances prediction performance for variants with limited data but also maintains high performance for larger datasets.

Additionally, the cross-variant training strategy eliminates the need to train separate models for each variant, significantly reducing overall training time. This approach achieves both improved performance and greater training efficiency, addressing the limitations of variant-specific training while enhancing both predictive accuracy and generalization capabilities.

### 3.2 Comparison with machine learning and deep learning baseline models

**Figure 3.**
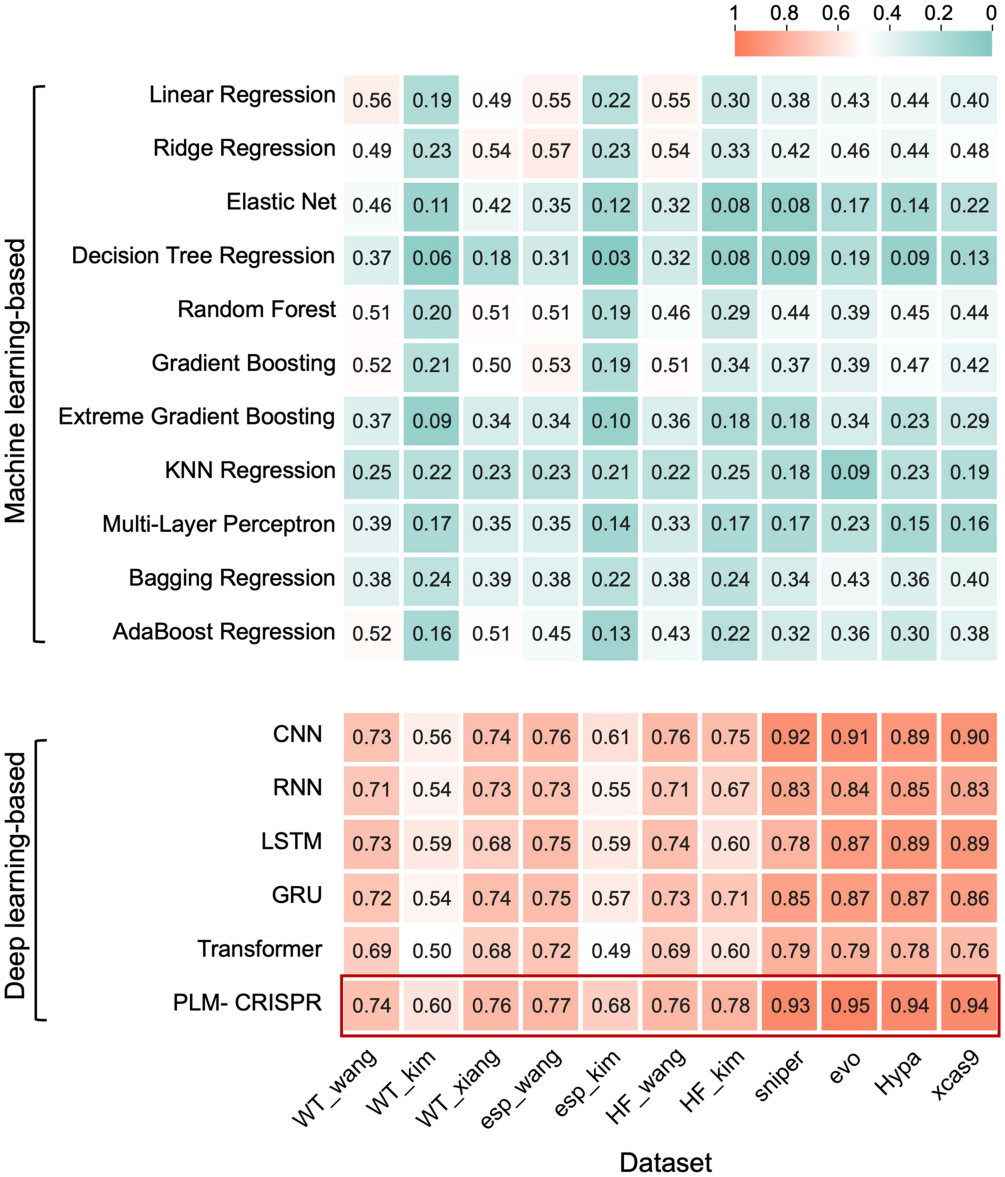
Heatmap of Spearman correlation coefficients for PLM-CRISPR compared with traditional machine learning baselines (top) and classical deep learning baselines (bottom).

To demonstrate the effectiveness of PLM-CRISPR over traditional machine learning models in predicting sgRNA activity, we conducted a comprehensive comparison against 11 machine learning baseline models, including linear regression, ridge regression, elastic net, decision tree regression, random forest, gradient boosting, extreme gradient boosting, *k*-nearest neighbor (KNN) regression, bagging regression, AdaBoost regression, and MLP. Since existing sgRNA activity prediction methods primarily rely on sgRNA sequence characteristics, we benchmarked PLM-CRISPR against the machine learning models using only sgRNA sequence-derived features. Specifically, sgRNA sequences were one-hot encoded to generate input features for training and prediction. All models were trained and tested on the same datasets and the consistent evaluation metric to ensure a fair comparison. As shown in Fig. 3 and Table 3, available as supplementary data at *Bioinformatics* online, these traditional machine learning models exhibit inferior performance in predicting sgRNA activity across different Cas9 protein (variants). In contrast, PLM-CRISPR consistently outperforms these traditional machine learning models, highlighting its ability to capture complex biological patterns.

To further validate the effectiveness of our model architecture, we compared PLM-CRISPR with five classical deep learning baseline models, including CNN, RNN, LSTM, GRU, and Transformer. Specifically, we replaced the sgRNA feature extraction module of PLM-CRISPR with each of these deep learning baseline models. As shown in Fig. 3 and Table 4, available as supplementary data at *Bioinformatics* online, PLM-CRISPR consistently outperforms all classic deep learning baseline models, demonstrating its unique capability to capture gene-editing-specific regulatory patterns. Although these classical deep learning models are highly effective for sequence modeling, they face challenges in variant-specific feature representation. Overall, PLM-CRISPR demonstrates significant advantages as a hybrid deep learning framework for sgRNA activity prediction.

### 3.3 Performance comparison of sgRNA prediction models across different application scenarios

In real-world applications, when a new Cas9 variant is discovered, there is typically insufficient experimental data on sgRNA activity to train an effective model. Therefore, to evaluate the performance of PLM-CRISPR in real-world applications with datasets of varying sizes, we simulated three distinct application scenarios based on the characteristics of the collected datasets. Specifically, we first categorized the 11 datasets according to their sample sizes. Datasets from variants of HypaCas9, SniperCas9, evoCas9, and xCas9 (with sample sizes of less than 1500) are classified as small-scale, newly identified datasets, while the remaining datasets are classified as large-scale, well-established datasets. Then, we designed three training and testing strategies to simulate and compare sgRNA activity prediction methods of different real-world scenarios: modeling for well-established, newly identified, and newly discovered Cas9 protein (variants).

**Figure 4.**
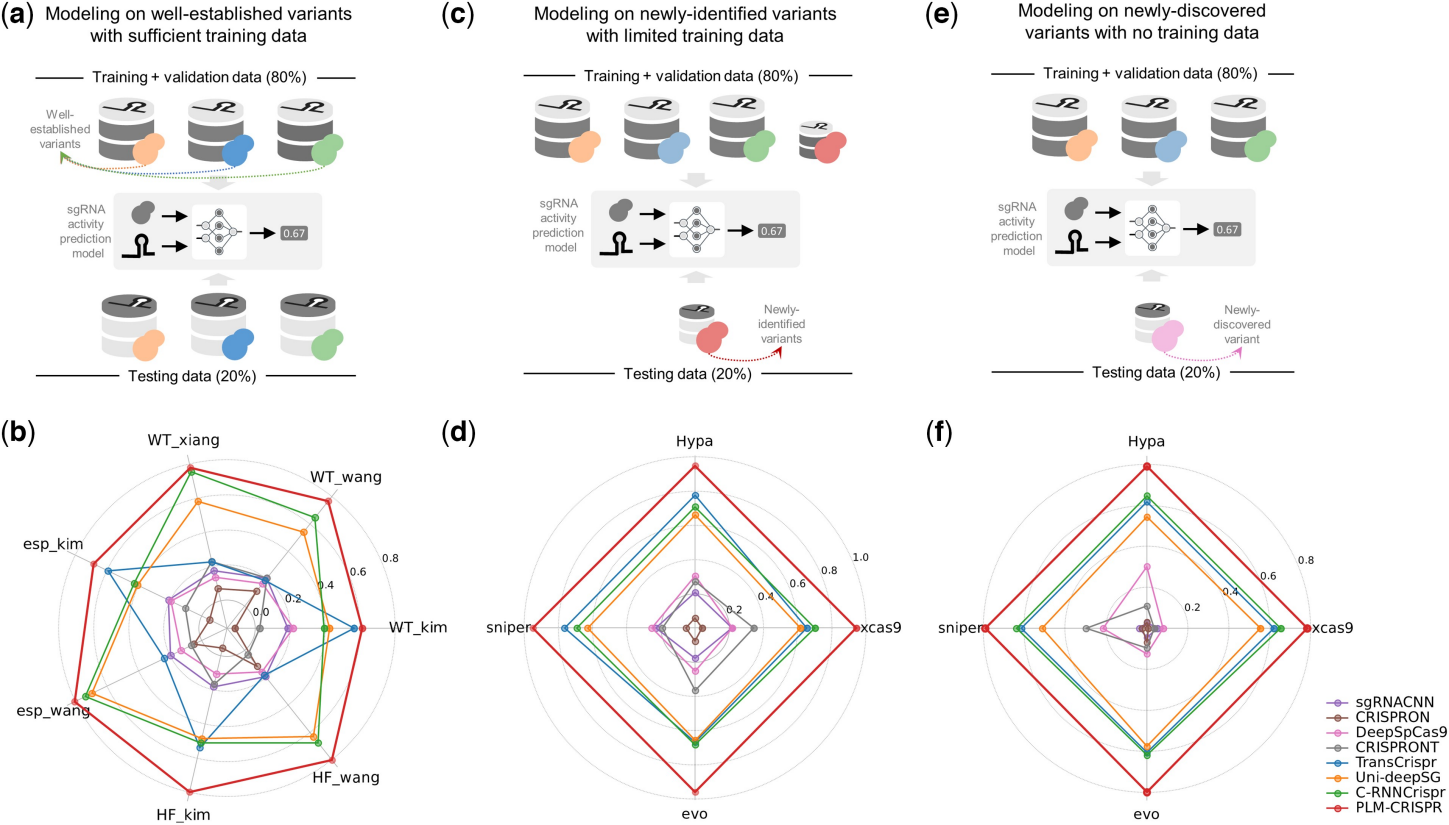
Schematic illustration and performance comparison of PLM-CRISPR with existing sgRNA activity prediction methods across three application scenarios. (a) Schematic of the well-established variant scenario, modeling on well-established variants with sufficient training data. (b) Spearman correlation coefficient comparisons in the simulated well-established variant scenario. (c) Schematic of the simulated newly identified variant scenario, involving variants with limited training data. (d) Spearman correlation coefficient comparisons in the newly identified variant scenario. (e) Schematic of the simulated newly discovered scenario, where testing is conducted on variants with no training data. (f) Spearman correlation coefficient comparisons in the newly discovered variant scenario.

Figure 4 illustrates the training and testing strategies across multiple variant datasets of varying scales for the three application scenarios. We comprehensively compared PLM-CRISPR with existing state-of-the-art sgRNA activity prediction methods under each of these scenarios, with all models trained from scratch for the specific needs of each scenario. The methods compared include sgRNACNN (Niu *et al.* 2021), CRISPRON (Xiang *et al.* 2021), DeepSpCas9 (Kim *et al.* 2019), C-RNNCrispr (Zhang *et al.* 2020), CRISPRONT (Zhang *et al.* 2021), TransCrispr (Wan and Jiang 2023), and Uni-deepSG (Zhong *et al.* 2023).

#### 3.3.1 Well-established variant scenario

In the well-established variant scenario, which aims to model an efficient predictor using large-scale, high-quality sgRNA activity data, we trained and tested the model using seven large-scale variant datasets (data splits detailed in Table 5, available as supplementary data at *Bioinformatics* online). As shown in Fig. 4b, PLM-CRISPR demonstrates exceptional performance across the seven datasets, achieving higher performance (e.g. datasets from Wang *et al.* (2019)) compared to smaller ones. This confirms PLM-CRISPR’s strong prediction power when data is abundant.

#### 3.3.2 Newly identified variant scenario

To evaluate PLM-CRISPR’s performance in scenarios involving newly identified Cas9 protein (variants) with limited labeled sgRNA activity data, we simulated the scenario. Specifically, we trained models using training sets from all variants, and tested models on datasets from four newly identified variants (HypaCas9, SniperCas9, evoCas9, and xCas9; data splitting detailed in Table 6, available as supplementary data at *Bioinformatics* online). As shown in Fig. 4d, PLM-CRISPR consistently outperforms all comparison methods on the four newly identified variant datasets, achieving an average Spearman correlation coefficient as high as 0.95. While several methods, such as TransCrispr, C-RNNCrispr, and Uni-deepSG perform well on certain variants (e.g. HypaCas9 and sniperCas9), their performance significantly drops on others. In contrast, other methods, such as sgRNACNN and CRISPRON, show limited performance, struggling with sgRNA activity prediction for Cas9 protein (variants) under data-scarce conditions. These results highlight PLM-CRISPR’s superior adaptability in the scenarios involving limited data for novel identified variants.

#### 3.3.3 Newly discovered variant scenario

To further evaluate PLM-CRISPR’s capability in scenarios where no training data are available for a newly discovered Cas9 variant, we simulated the scenario. Specifically, we trained models using training sets from the well-established variants and then directly tested on data from four small-scale variant datasets (HypaCas9, SniperCas9, evoCas9, and xCas9), without using any data from these variants during training. To ensure consistency, we kept the same test samples as in the “newly identified variant scenario” for comparison (data splits are detailed in Table 7, available as supplementary data at *Bioinformatics* online). As shown in Fig. 4f, the performance of most methods declines significantly when predicting sgRNA activity for newly discovered variants. In contrast, PLM-CRISPR still maintains a significant advantage (slightly worse than in the “newly identified variant scenario”), delivering the best prediction performance across all datasets. While some comparison methods, such as TransCrispr, Uni-deepSG, and C-RNNCrispr, demonstrate relatively stable predictions, their overall performance remains lower than that of PLM-CRISPR. The remaining models, including sgRNACNN and CRISPRON, struggle to provide powerful predictions, highlighting their limitations in data-scarce scenarios.

Across the three real-world scenarios—predicting sgRNA activity for well-established, newly identified, and newly discovered Cas9 protein (variants)— PLM-CRISPR consistently demonstrates superior prediction performance. These results highlight its exceptional adaptability and robustness, making it a highly effective tool for predicting sgRNA activity in novel Cas9 protein (variants), even under conditions of limited or no training data.

### 3.4 Motif analysis

To investigate whether PLM-CRISPR can distinguish sgRNA sequence patterns associated with high- and low-activity across different variants, we performed motif analysis. For each dataset, sgRNAs were categorized into high- and low-activity groups based on experimentally measured and predicted activity scores. Given the asymmetric and skewed distribution of activity values observed across datasets (as shown in Fig. 2, available as supplementary data at *Bioinformatics* online), we used the median as a threshold for more balanced and robust grouping. Specifically, sgRNAs with activity values above the median were categorized as high-activity, while those below the median were defined as low-activity. Motif analysis was performed using the pLogo generator tool (O’shea *et al.* 2013) to identify nucleotide enrichment and depletion patterns of high-activity sgRNAs compared to low-activity sgRNAs. In the analysis, we set the high-activity sgRNAs as the foreground dataset, while the low-activity sgRNAs as the background dataset, and generated enriched motif patterns for both experimental measured and PLM-CRISPR predicted sgRNA activity scores across seven variants.

**Figure 5.**
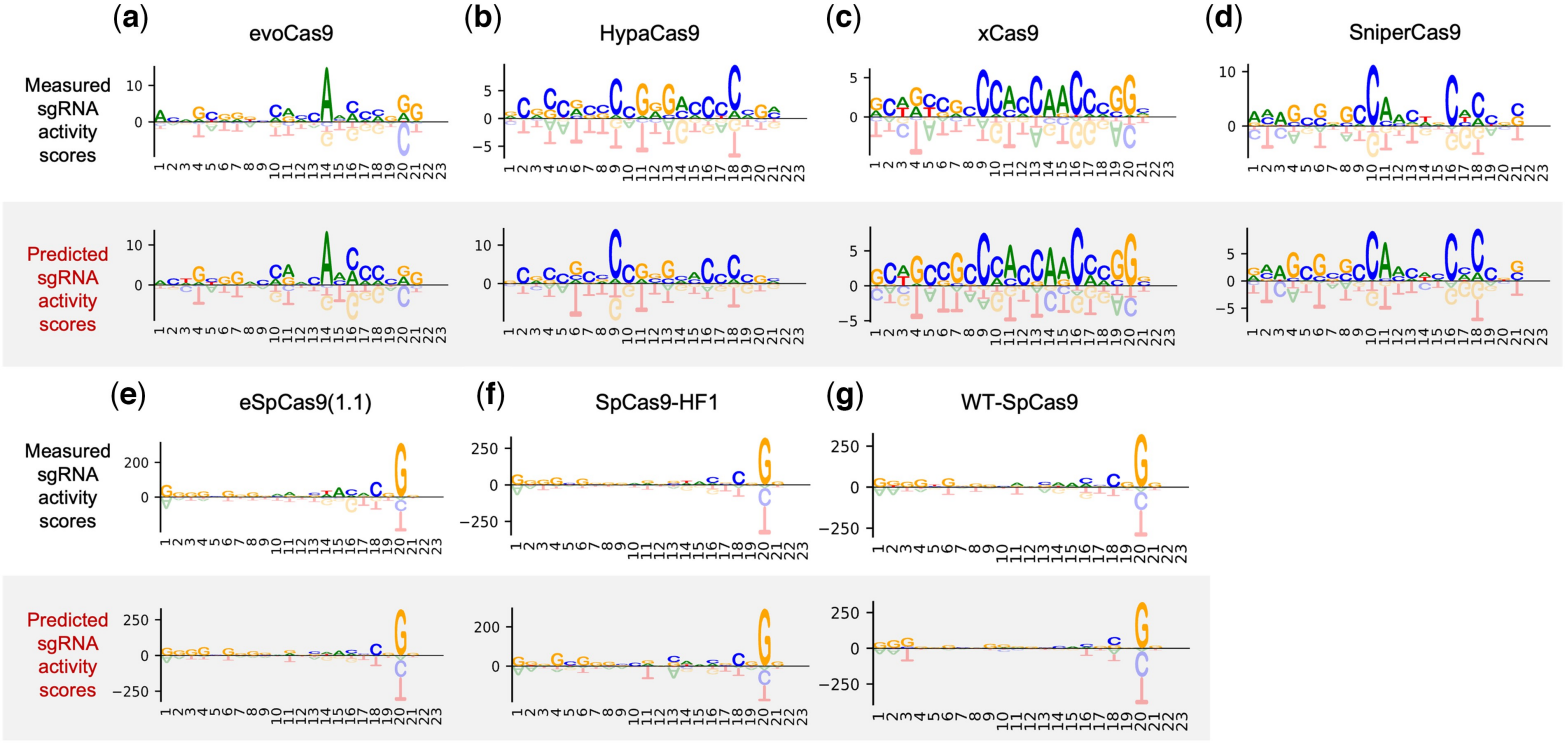
Motif enrichment analysis of high-activity and low-activity sgRNAs based on experimental measured sgRNA activity scores (top) and PLM-CRISPR-predicted sgRNA activity scores (bottom) across different Cas9 protein (variants). (a) evoCas9. (b) HypaCas9. (c) xCas9. (d) SniperCas9. (e) eSpCas9(1,1). (f) SpCas9-HF1. (g) WT-SpCas9.

As shown in Fig. 5, we can observe that PLM-CRISPR is able to capture nucleotide enrichment and depletion patterns highly consistently compared to the experimental measured data across multiple variants. This indicates that PLM-CRISPR can effectively distinguish high-activity and low-activity sgRNAs in different variants, demonstrating the model's robustness and efficiency in sgRNA activity prediction. Moreover, these patterns reveal a consistent trend across seven variants, with notable enrichment of G and C nucleotides, while the representation of A and T is relatively lower in these regions. The sgRNA with high GC content appears to contribute to binding stability, may provide value insights for designing sgRNAs with high activity and target specificity. Additionally, different variants exhibit specific patterns and diverse enriched nucleotide regions, which suggests that high-activity sgRNAs in different variants may have preferences for specific target binding sequences, which are reflected at specific positions.

### 3.5 Biological feature importance analysis

To further investigate why integrating multiple dataset training is beneficial for sgRNA activity prediction of the newly identified/discovered variant datasets with small sample sizes, we performed the TreeSHAP (Lundberg *et al.* 2019) analysis to measure the impact of varying sgRNA sequence features in different datasets. Specifically, we manually extracted 175 sgRNA sequence features based on prior biological knowledge and previous studies on sgRNA efficacy. These features were grouped into several categories, including GC content, single nucleotide compositions, dinucleotide compositions, trinucleotide compositions, nucleotide specific positions, and melting temperature ($T_{m}$). The $T_{m}$ was calculated using the nearest neighbor-based method (SantaLucia and Turner 1997). A comprehensive list and detailed description of all 175 features is provided in Table 8, available as supplementary data at *Bioinformatics* online. Then, we trained a XGBoost model on each dataset and used it to compute feature importance scores, allowing us to assess how the predictive value of each feature varies across different variant dataset. As can be seen from Fig. 3, available as supplementary data at *Bioinformatics* online, the importance of features captured of different datasets is significantly affected by sample size. Larger sample sizes (from eSpCas9(1.1), WT-SpCas9, and SpCas9-HF1 datasets) provide insight into the sensitivity for nucleotides at different positions of sgRNAs in the prediction model, while model trained from smaller sample sizes mainly focus on features of nucleotide compositions. Notably, TT dimers and AAA trimers exhibit significant importance across all datasets, whereas the importance of Pos_20: G (G at position 20) and Pos_20: C (C at position 20)—the nucleotides immediately adjacent to the PAM sequence—is only learned in larger sample datasets. This partly explains why the cross-variant strategy facilitates sgRNA activity prediction for newly identified/discovered variants, as it allows the model to leverage key position-specific information learned from well-established variant datasets to enhance predictions for novel variants. Additionally, GC content and melting temperature ($T_{m})$ also show significant importance across all datasets, consistent with our previous motif analysis and findings that have been reported (Xiang *et al.* 2017).

## 4 Conclusion

In this study, we proposed PLM-CRISPR, a unified deep learning framework for predicting sgRNA activity across multiple Cas9 protein (variants). PLM-CRISPR offers three key advantages:

1. Unlike existing methods that focus solely on sgRNA sequences, PLM-CRISPR integrates both sgRNA and Cas9 variant sequences, which simulate complex editing systems, enabling accurate sgRNA activity prediction.
2. The cross-variant training strategy significantly improves the model’s generalization ability, making it a robust model for predicting sgRNA activity even in scenarios with limited data.
3. By leveraging advanced pre-trained PLM ESM2 and multi-scale TextCNN, PLM-CRISPR effectively captures variant and sgRNA representations, enhancing prediction performance.

Extensive experiments demonstrate that PLM-CRISPR outperforms existing methods across 11 datasets spanning seven Cas9 protein (variants). While some methods rely on variant-specific training to achieve high performance, their generalization abilities are limited. In contrast, PLM-CRISPR's cross-variant training strategy ensures excellent generalization and robustness, enabling accurate prediction of sgRNA activity in different data scales and complex gene-editing scenarios. Notably, PLM-CRISPR not only predicts sgRNA activity for known variants but also extends its capabilities to unidentified variants, thereby broadening the applicability of CRISPR/Cas9 technology.

Despite the advantages demonstrated by PLM-CRISPR, some limitations remain. First, while the model effectively handles a range of Cas9 protein (variants), its performance is still be constrained by the quality and scope of training data. Second, the sequence-based protein representation method used in PLM-CRISPR cannot capture the structural and functional differences between variants, thus limiting its prediction performance. Future work could address these limitations by incorporating protein structural information to better capture variant-specific properties and further enhance predictions for novel variants.

## Supplementary Material

btaf385_Supplementary_Data

## Data Availability

All data used in this study are available at https://github.com/CSUBioGroup/PLM-CRISPR.
