# Supplementary material for: Leveraging protein language models for cross-variant CRISPR/Cas9 sgRNA activity prediction
Source: Bioinformatics. 2025 Jul 2;41(7):btaf385. doi: 10.1093/bioinformatics/btaf385 (PMC12254127; doi:10.1093/bioinformatics/btaf385)
Supplement: btaf385_Supplementary_Data [file btaf385_supplementary_data.pdf]

Supplementary Materials

Leveraging protein language models for cross-variant CRISPR/Cas9 sgRNA activity prediction

Yalin Hou[1, #], Yiming Li[1, #], Ruiqing Zheng[1], Fuhao Zhang[2], Fei Guo[1], Min Li[1], Min Zeng[1, *]

[1]School of Computer Science and Engineering, Central South University, Changsha, 410083, China

[2]College of Information Engineering, Northwest A&F University, Yangling, Shanxi, 712100, China

[#] These authors contributed equally to this work

* Correspondence to: Min Zeng, E-mail: zengmin@csu.edu.cn

This supplementary file includes:

1. Supplementary Tables

Supplementary Table S1. Structural domains involved in the key mutation points of Cas variants and their function information.

Supplementary Table S2. Summary of mathematical symbols and their definitions.

Supplementary Table S3. Comparative performance of PLM-CRISPR and traditional machine learning models in predicting sgRNA activity across Cas9 variants.

Supplementary Table S4. Comparative performance of PLM-CRISPR and deep learning baseline models in predicting sgRNA activity across Cas9 variants.

Supplementary Table S5. Data segmentation in the well-established

variant scenario.

Supplementary Table S6. Data segmentation in the newly identified variant scenario.

Supplementary Table S7. Data segmentation in the newly discovered variant scenario.

Supplementary Table S8. Detailed information on the 175 manually derived sgRNA sequence features.

2. Supplementary Figures

Supplementary Figure. S1. Histograms of sgRNA activity scores for the training sets (left), validation sets (middle), and test sets (right) across all datasets.

Supplementary Figure. S2. Statistical distribution of experimentally measured sgRNA activity scores across the seven variant datasets.

Supplementary Figure. S3. TreeSHAP analysis of sgRNA sequence feature importance across diverse Cas9 variants. (a) evoCas9. (b) HypaCas9. (c) SniperCas9. (d) xCas9. (e) eSpCas9(1,1). (f) SpCas9-HF1. (g) WT-SpCas9.

1. Supplementary Tables

Supplementary Table S1. Structural domains involved in the key mutation points of Cas variants and their function information.

Domain	Function	Key Mutations	Influence	Corresponding Variants
RuvC3 (909-1098)	Effects on non-target strand cleavage and sgRNA-mediated DNA recognition	Q926A	Optimizing RuvC3 cleavage specificity and reducing off-target effects	SpCas9-HF1
		K1003A, R1060A	Reduces RuvC3 mis-cleavage and improves DNA recognition specificity	eSpCas9(1.1)
REC3 (268-713)	Effects on sgRNA binding stability and HNH activation	N497A, R661A, Q695A	Reduce off-target effects and improve editing specificity	SpCas9-HF1
		F539S	Improve DNA binding capacity	SniperCas9
		M495V, Y515N, K526E, R661Q	Influence DNA binding stability and optimize off-target properties	evoCas9
		R324L, S409I, E480K, E543D, M694I	Affects protein structural stability and the way Cas9 binds to target DNA	xCas9
		N692A, M694A, Q695A, H698A	Improve editing accuracy	HypaCas9
HNH (775-908)	Effects on target strand cleavage and conformational transitions of the Cas9 complex	K848A	Influence HNH activation and improve specificity	eSpCas9(1.1)
		K890N	Directly affects target chain cutting	SniperCas9

Supplementary Table S2. Summary of mathematical symbols and their definitions.

Equation Symbol	Definition
$X_{RNA} \in \mathbb{R}^{L\times D}$	The input matrix of sgRNA sequence
L	The length of the input sequence
D	The embedding dimension of the input
i	The output position index
k	The index of the filter
M	The window size
N	The number of input channels
W_k	The convolutional filter
b^k	The bias of the k-th convolution kernel
σ	The Sigmoid activation function
l	The l-th convolutional layer
Z^l	The output features of the l-th layer
$X_{pt} \in \mathbb{R}^{L\times D}$	The input matrix of protein sequences
j	The scale index
$M_j \in \{5,9,13\}$	The size of the convolution kernel of the j-th scale
H_{RNA}	The output of the sgRNA sequence feature extraction module
H_{pt}	The output of the protein variant sequence feature extraction module
ρ	Spearman correlation coefficient
I	The total number of observations
d_i	The rank difference of the i-th data point between the two variables
w	The weight of feature vector

Supplementary Table S3. Comparative performance of PLM-CRISPR and traditional machine learning models in predicting sgRNA activity across Cas9 variants.

Model	WT_wang	WT_kim	WT_xiang	esp_wang	esp_kim	HF_wang	HF_kim	sniper	evo	Hypa	xcas9
Linear Regression	0.560	0.186	0.494	0.55	0.224	0.545	0.297	0.380	0.427	0.443	0.401
Ridge Regression	0.495	0.231	0.538	0.571	0.235	0.537	0.325	0.422	0.460	0.436	0.480
Elastic Net	0.460	0.111	0.423	0.352	0.122	0.324	0.083	0.082	0.175	0.14	0.221
Decision Tree Regression	0.367	0.059	0.184	0.310	0.033	0.315	0.079	0.088	0.194	0.085	0.133
Random Forest	0.512	0.198	0.513	0.508	0.193	0.464	0.294	0.442	0.390	0.451	0.438
Gradient Boosting	0.516	0.209	0.501	0.532	0.190	0.512	0.338	0.373	0.387	0.466	0.417
Extreme Gradient Boosting	0.367	0.086	0.337	0.339	0.095	0.356	0.181	0.181	0.338	0.231	0.290
KNN Regression	0.254	0.217	0.230	0.227	0.213	0.223	0.252	0.185	0.094	0.225	0.194
Multi-Layer Perceptron	0.393	0.170	0.353	0.355	0.135	0.326	0.171	0.170	0.229	0.152	0.156
Bagging Regression	0.376	0.241	0.386	0.380	0.223	0.378	0.236	0.342	0.435	0.365	0.399
AdaBoost Regression	0.523	0.156	0.506	0.452	0.129	0.435	0.221	0.323	0.358	0.301	0.380
**PLM-CRISPR**	**0.736**	**0.605**	**0.757**	**0.766**	**0.681**	**0.763**	**0.778**	**0.933**	**0.955**	**0.935**	**0.936**

Supplementary Table S4. Comparative performance of PLM-CRISPR and deep learning baseline models in predicting sgRNA activity across Cas9 variants.

Model	WT_wang	WT_kim	WT_xiang	esp_wang	esp_kim	HF_wang	HF_kim	sniper	evo	Hypa	xcas9
CNN	0.729	0.565	0.743	0.759	0.614	0.759	0.749	0.916	0.913	0.892	0.904
RNN	0.711	0.543	0.729	0.727	0.549	0.713	0.673	0.833	0.842	0.855	0.831
LSTM	0.734	0.587	0.680	0.752	0.586	0.738	0.603	0.776	0.871	0.889	0.887
GRU	0.722	0.540	0.739	0.747	0.570	0.731	0.710	0.852	0.867	0.868	0.857
Transformer	0.687	0.499	0.683	0.721	0.494	0.695	0.599	0.789	0.788	0.775	0.757
**PLM-CRISPR**	**0.736**	**0.605**	**0.757**	**0.766**	**0.681**	**0.763**	**0.778**	**0.933**	**0.955**	**0.935**	**0.936**

Supplementary Table S5. Data segmentation in the well-established variant scenario.

Category	Dataset Name	Protein Variants	Training Samples	Validation Samples	Testing Samples	Total
Large	WT_kim	WT-SpCas9	3884	553	1109	5546
Large	HF_kim	SpCas9-HF1	3935	564	1132	5631
Large	esp_kim	eSpCas9 （1.1）	3952	567	1129	5648
Large	WT_wang	WT-SpCas9	32246	4606	9246	46098
Large	HF_wang	SpCas9-HF1	32975	4711	9473	47159
Large	esp_wang	eSpCas9 （1.1）	34030	4844	9723	48597
Large	WT_xiang	WT-SpCas9	6188	884	1768	8840

Supplementary Table S6. Data segmentation in the newly identified variant scenario.

Category	Dataset Name	Protein Variants	Training Samples	Validation Samples	Testing Samples	Total
Large	WT_kim	WT-SpCas9	3884	553	0	4437
Large	HF_kim	SpCas9-HF1	3935	564	0	4499
Large	esp_kim	eSpCas9 （1.1）	3952	567	0	4519
Large	WT_wang	WT-SpCas9	32246	4606	0	36852
Large	HF_wang	SpCas9-HF1	32975	4711	0	37686
Large	esp_wang	eSpCas9 （1.1）	34030	4844	0	38874
Large	WT_xiang	WT-SpCas9	6188	884	0	7072
Small	Hypa	HypaCas9	823	117	235	1175
Small	sniper	SniperCas9	963	137	275	1375
Small	evo	evoCas9	949	135	271	1355
Small	xcas9	xCas9	955	137	273	1365

Supplementary Table S7. Data segmentation in the newly discovered variant scenario.

Category	Dataset Name	Protein Variants	Training Samples	Validation Samples	Testing Samples	Total
Large	WT_kim	WT-SpCas9	3884	553	0	4437
Large	HF_kim	SpCas9-HF1	3935	564	0	4499
Large	esp_kim	eSpCas9 （1.1）	3952	567	0	4519
Large	WT_wang	WT-SpCas9	32246	4606	0	36852
Large	HF_wang	SpCas9-HF1	32975	4711	0	37686
Large	esp_wang	eSpCas9 （1.1）	34030	4844	0	38874
Large	WT_xiang	WT-SpCas9	6188	884	0	7072
Small	Hypa	HypaCas9	0	0	235	235
Small	sniper	SniperCas9	0	0	275	275
Small	evo	evoCas9	0	0	271	271
Small	xcas9	xCas9	0	0	273	273

Supplementary Table S8. Detailed information on the 175 manually derived sgRNA sequence features.

Feature Name	Definitions	Category (Number)
GC_Content	Nucleotide G and C content	GC content (1)
Tm	Melting temperature of the full sgRNA sequence	Melting temperature (4)
Tm_1_4	Melting temperature of nucleotides at positions 1 to 4	
Tm_5_15	Melting temperature of nucleotides at positions 5 to 15	
Tm_16_20	Melting temperature of nucleotides at positions 16 to 20	
Count_A	Counting of nucleotide A	Single nucleotide compositions (4)
Count_G	Counting of nucleotide G	
Count_C	Counting of nucleotide C	
Count_T	Counting of nucleotide T	
Pos_1:A	Nucleotide type A at the first position	Nucleotide positions (86)
Pos_1:G	Nucleotide type G at the first position	
Pos_1:C	Nucleotide type C at the first position	
Pos_1:T	Nucleotide type T at the first position	
Pos_2:A	Nucleotide type A at the second position	
Pos_2:G	Nucleotide type G at the second position	
Pos_2:C	Nucleotide type C at the second position	
Pos_2:T	Nucleotide type T at the second position	
Pos_3:A	Nucleotide type A at the third position	
Pos_3:G	Nucleotide type G at the third position	
Pos_3:C	Nucleotide type C at the third position	
Pos_3:T	Nucleotide type T at the third position	
Pos_4:A	Nucleotide type A at the fourth position	
Pos_4:G	Nucleotide type G at the fourth position	
Pos_4:C	Nucleotide type C at the fourth position	
Pos_4:T	Nucleotide type T at the fourth position	
Pos_5:A	Nucleotide type A at the fifth position	
Pos_5:G	Nucleotide type G at the fifth position	
Pos_5:C	Nucleotide type C at the fifth position	
Pos_5:T	Nucleotide type T at the fifth position	
Pos_6:A	Nucleotide type A at the sixth position	
Pos_6:G	Nucleotide type G at the sixth position	
Pos_6:C	Nucleotide type C at the sixth position	
Pos_6:T	Nucleotide type T at the sixth position	
Pos_7:A	Nucleotide type A at the seventh position	
Pos_7:G	Nucleotide type G at the seventh position	
Pos_7:C	Nucleotide type C at the seventh position	
Pos_7:T	Nucleotide type T at the seventh position	
Pos_8:A	Nucleotide type A at the eighth position	
Pos_8:G	Nucleotide type G at the eighth position	
Pos_8:C	Nucleotide type C at the eighth position	
Pos_8:T	Nucleotide type T at the eighth position	
Pos_9:A	Nucleotide type A at the ninth position	

Pos_9:G	Nucleotide type G at the ninth position
Pos_9:C	Nucleotide type C at the ninth position
Pos_9:T	Nucleotide type T at the ninth position
Pos_10:A	Nucleotide type A at the tenth position
Pos_10:G	Nucleotide type G at the tenth position
Pos_10:C	Nucleotide type C at the tenth position
Pos_10:T	Nucleotide type T at the tenth position
Pos_11:A	Nucleotide type A at the eleventh position
Pos_11:G	Nucleotide type G at the eleventh position
Pos_11:C	Nucleotide type C at the eleventh position
Pos_11:T	Nucleotide type T at the eleventh position
Pos_12:A	Nucleotide type A at the twelfth position
Pos_12:G	Nucleotide type G at the twelfth position
Pos_12:C	Nucleotide type C at the twelfth position
Pos_12:T	Nucleotide type T at the twelfth position
Pos_13:A	Nucleotide type A at the thirteenth position
Pos_13:G	Nucleotide type G at the thirteenth position
Pos_13:C	Nucleotide type C at the thirteenth position
Pos_13:T	Nucleotide type T at the thirteenth position
Pos_14:A	Nucleotide type A at the fourteenth position
Pos_14:G	Nucleotide type G at the fourteenth position
Pos_14:C	Nucleotide type C at the fourteenth position
Pos_14:T	Nucleotide type T at the fourteenth position
Pos_15:A	Nucleotide type A at the fifteenth position
Pos_15:G	Nucleotide type G at the fifteenth position
Pos_15:C	Nucleotide type C at the fifteenth position
Pos_15:T	Nucleotide type T at the fifteenth position
Pos_16:A	Nucleotide type A at the sixteenth position
Pos_16:G	Nucleotide type G at the sixteenth position
Pos_16:C	Nucleotide type C at the sixteenth position
Pos_16:T	Nucleotide type T at the sixteenth position
Pos_17:A	Nucleotide type A at the seventeenth position
Pos_17:G	Nucleotide type G at the seventeenth position
Pos_17:C	Nucleotide type C at the seventeenth position
Pos_17:T	Nucleotide type T at the seventeenth position
Pos_18:A	Nucleotide type A at the eighteenth position
Pos_18:G	Nucleotide type G at the eighteenth position
Pos_18:C	Nucleotide type C at the eighteenth position
Pos_18:T	Nucleotide type T at the eighteenth position
Pos_19:A	Nucleotide type A at the nineteenth position
Pos_19:G	Nucleotide type G at the nineteenth position
Pos_19:C	Nucleotide type C at the nineteenth position
Pos_19:T	Nucleotide type T at the nineteenth position
Pos_20:A	Nucleotide type A at the twentieth position
Pos_20:G	Nucleotide type G at the twentieth position
Pos_20:C	Nucleotide type C at the twentieth position

Pos_20:T	Nucleotide type T at the twentieth position	
Pos_21:A	Nucleotide type A at the twenty-first position	
Pos_21:G	Nucleotide type G at the twenty-first position	
Pos_21:C	Nucleotide type C at the twenty-first position	
Pos_21:T	Nucleotide type T at the twenty-first position	
Pos_22:G	Nucleotide type G at the twenty-second position	
Pos_23:G	Nucleotide type G at the twenty-third position	
AA	Counting of the dinucleotide combination AA	
AC	Counting of the dinucleotide combination AC	
AG	Counting of the dinucleotide combination AG	
AT	Counting of the dinucleotide combination AT	
CA	Counting of the dinucleotide combination CA	
CC	Counting of the dinucleotide combination CC	
CG	Counting of the dinucleotide combination CG	
CT	Counting of the dinucleotide combination CT	Dinucleotide compositions (16)
GA	Counting of the dinucleotide combination GA	
GC	Counting of the dinucleotide combination GC	
GG	Counting of the dinucleotide combination GG	
GT	Counting of the dinucleotide combination GT	
TA	Counting of the dinucleotide combination TA	
TC	Counting of the dinucleotide combination TC	
TG	Counting of the dinucleotide combination TG	
TT	Counting of the dinucleotide combination TT	
AAA	Counting of the trinucleotide combination AAA	
AAC	Counting of the trinucleotide combination AAC	
AAG	Counting of the trinucleotide combination AAG	
AAT	Counting of the trinucleotide combination AAT	
ACA	Counting of the trinucleotide combination ACA	
ACC	Counting of the trinucleotide combination ACC	
ACG	Counting of the trinucleotide combination ACG	
ACT	Counting of the trinucleotide combination ACT	
AGA	Counting of the trinucleotide combination AGA	
AGC	Counting of the trinucleotide combination AGC	
AGG	Counting of the trinucleotide combination AGG	
AGT	Counting of the trinucleotide combination AGT	Trinucleotide compositions (64)
ATA	Counting of the trinucleotide combination ATA	
ATC	Counting of the trinucleotide combination ATC	
ATG	Counting of the trinucleotide combination ATG	
ATT	Counting of the trinucleotide combination ATT	
CAA	Counting of the trinucleotide combination CAA	
CAC	Counting of the trinucleotide combination CAC	
CAG	Counting of the trinucleotide combination CAG	
CAT	Counting of the trinucleotide combination CAT	
CCA	Counting of the trinucleotide combination CCA	
CCC	Counting of the trinucleotide combination CCC	
CCG	Counting of the trinucleotide combination CCG	

CCT	Counting of the trinucleotide combination CCT
CGA	Counting of the trinucleotide combination CGA
CGC	Counting of the trinucleotide combination CGC
CGG	Counting of the trinucleotide combination CGG
CGT	Counting of the trinucleotide combination CGT
CTA	Counting of the trinucleotide combination CTA
CTC	Counting of the trinucleotide combination CTC
CTG	Counting of the trinucleotide combination CTG
CTT	Counting of the trinucleotide combination CTT
GAA	Counting of the trinucleotide combination GAA
GAC	Counting of the trinucleotide combination GAC
GAG	Counting of the trinucleotide combination GAG
GAT	Counting of the trinucleotide combination GAT
GCA	Counting of the trinucleotide combination GCA
GCC	Counting of the trinucleotide combination GCC
GCG	Counting of the trinucleotide combination GCG
GCT	Counting of the trinucleotide combination GCT
GGA	Counting of the trinucleotide combination GGA
GGC	Counting of the trinucleotide combination GGC
GGG	Counting of the trinucleotide combination GGG
GGT	Counting of the trinucleotide combination GGT
GTA	Counting of the trinucleotide combination GTA
GTC	Counting of the trinucleotide combination GTC
GTG	Counting of the trinucleotide combination GTG
GTT	Counting of the trinucleotide combination GTT
TAA	Counting of the trinucleotide combination TAA
TAC	Counting of the trinucleotide combination TAC
TAG	Counting of the trinucleotide combination TAG
TAT	Counting of the trinucleotide combination TAT
TCA	Counting of the trinucleotide combination TCA
TCC	Counting of the trinucleotide combination TCC
TCG	Counting of the trinucleotide combination TCG
TCT	Counting of the trinucleotide combination TCT
TGA	Counting of the trinucleotide combination TGA
TGC	Counting of the trinucleotide combination TGC
TGG	Counting of the trinucleotide combination TGG
TGT	Counting of the trinucleotide combination TGT
TTA	Counting of the trinucleotide combination TTA
TTC	Counting of the trinucleotide combination TTC
TTG	Counting of the trinucleotide combination TTG
TTT	Counting of the trinucleotide combination TTT

2. Supplementary Figures

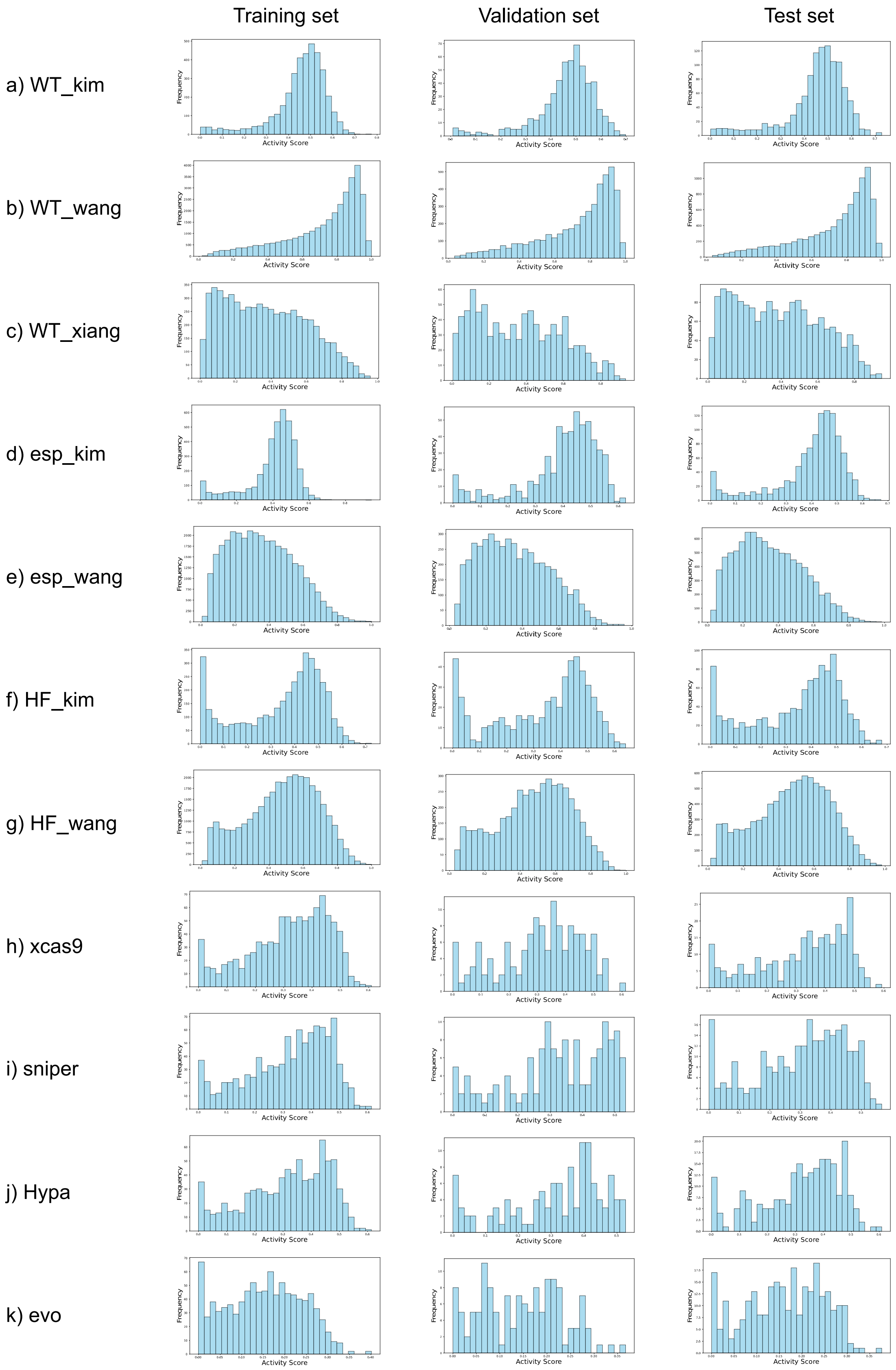

Supplementary Figure. S1. Histograms of sgRNA activity scores for the training sets (left), validation sets (middle), and test sets (right) across all datasets.

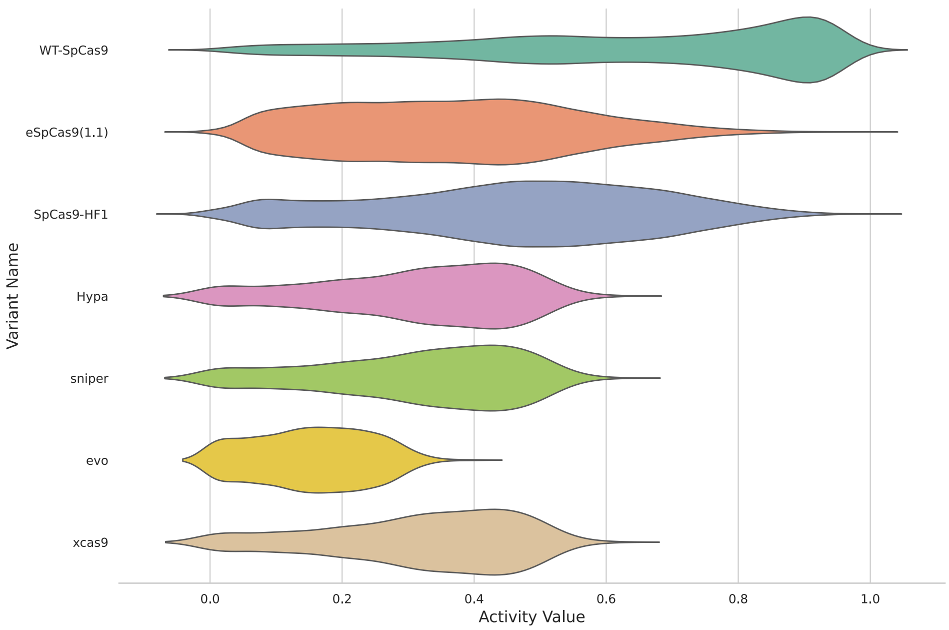

Supplementary Figure. S2. Statistical distribution of experimentally measured sgRNA activity scores across the seven variant datasets.

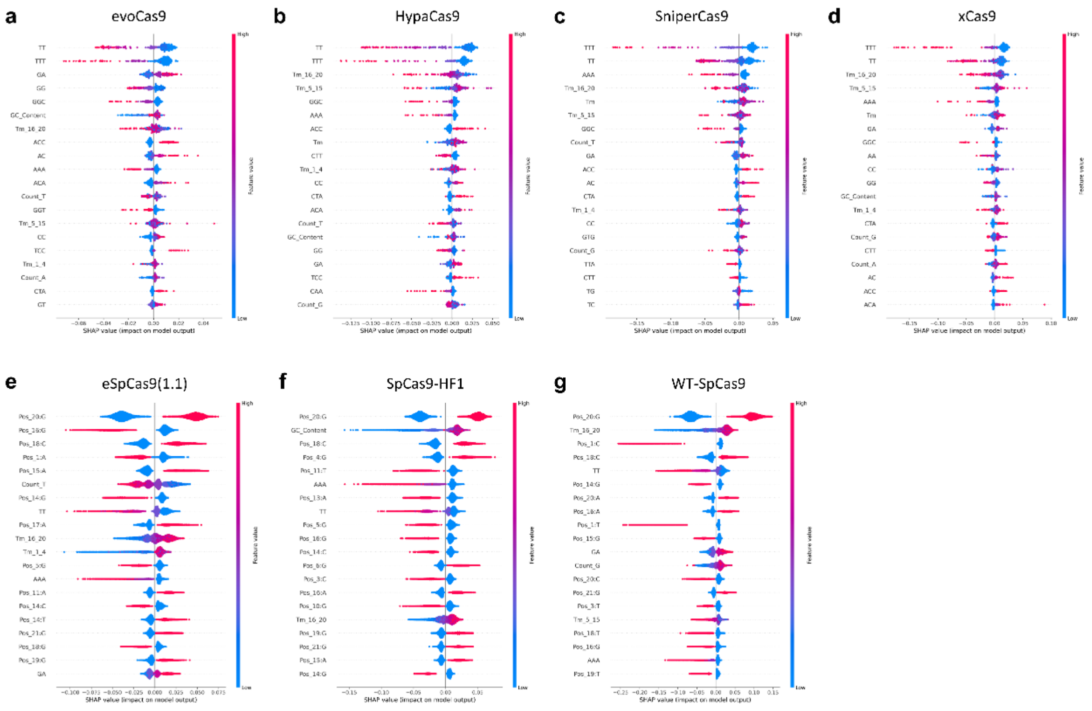

Supplementary Figure S3. TreeSHAP analysis of sgRNA sequence feature importance across diverse Cas9 variants. (a) evoCas9. (b) HypaCas9. (c) SniperCas9. (d) xCas9. (e) eSpCas9(1,1). (f) SpCas9-HF1. (g) WT-SpCas9.
